# Autobiographical Memory, Personality, and Facebook Mementos

**DOI:** 10.5964/ejop.v15i3.1713

**Published:** 2019-09-27

**Authors:** Barbara Caci, Maurizio Cardaci, Silvana Miceli

**Affiliations:** a Department of Psychology, Educational Science, and Human Movement, University of Palermo, Palermo, Italy; Connection Lab, San Francisco, CA, USA

**Keywords:** autobiographical memory, personality, Big Five, Facebook, social network sites

## Abstract

The present study analyzed the relationships between directive, self and social functions of autobiographical memory, personality traits, as defined by the Five Factor model, and the Facebook mementos. We defined Facebook mementos as objective measures of the textual (i.e., Facebook Status Updating) and visual (i.e., Photos uploading) information people record on their Facebook profiles. Questionnaires gathered data from a sample of 193 Italian Facebook users (148 female; 45 male; age M = 22.8, SD = 6.8). Results at path analysis using AMOS showed direct significant positive associations between personality traits related to extraversion, openness, neuroticism, conscientiousness and Facebook mementos. Extraversion and openness were positive precursors of the directive, self and social functions of autobiographical memory, whereas neuroticism predicted directive and self-functions, and conscientiousness was a positive precursor of the directive function of autobiographical memory. As well, indirect significant positive paths among extraversion, neuroticism, openness and the frequency of photos uploaded on Facebook for collecting life events have emerged via the mediation of the self-continuity function of autobiographical memory. In sum, the present study highlights how individual differences in motivations for using autobiographical memory for directive-behavior, self-continuity or social-bonding purposes deeply related with the personal experience of using social media as a repository tool for textual or visual information.

Autobiographical Memories (AMs) are long-term memories, defined by psychologists as recollections of the first-person experience of past episodes that refer to spatially and temporally specific life events rather than to semantic knowledge about the world (Bayley, Hopkins, & Squire, 2006; Manns, Hopkins, & Squire, 2003; Remondes & Schuman, 2004; Squire, Knowlton, & Musen, 1993). AMs are memories in which individual events that occurred throughout the life guides self-building and personal identity (Seyfi & Soydaş, 2017), and represents a link between what people have been in the past, what are in the present, and what they want to be in the future (e.g., Schacter, 1996). Strictly related to the life story of a person, AMs also connected with dispositional personality traits (McAdams, 2001; McAdams & Pals, 2006). Specifically, the different directive, self and social functions of AMs (Bluck, 2003; Cohen, 1998; Pillemer, 1992) modulate individual differences (Er & Yaşın, 2016). Nowadays, people use social networking sites (SNS) for collecting and sharing memories. For instance, on Facebook individuals edit and manage different kind of textual or visual memories about themselves and their life events (Back et al., 2010; Boyd, 2009; Nosko, Wood, & Molema, 2010). In this paper, we specifically focus on the relationships between AMs, personality traits and Facebook mementos considering that to date (on our knowledge), little studies have analyzed the relationships between these variables. Hence, this paper aims to fill to this gap, also examining the predictive role of personality traits on Facebook mementos through the mediation of directive, self and social functions of AMs (e.g., Bluck, 2003). The study of such relationships might provide, first, an empirical evaluation of the universality of the traditional functional approach to AMs when we applied it to personal life events posted online (e.g., Alea & Bluck, 2007; Baddeley, 1988; Baddeley & Singer, 2008; Fivush, 1998; Kulkofsky, Wang, & Hou, 2010; Nelson, 1993; Rasmussen & Berntsen, 2009). Second, we might explore whether and how social media represent a virtual extension of our memories (e.g., Lee, Im, & Taylor, 2008; Lenhart & Fox, 2006; Wang, Lee, & Hou, 2017).

## A Literature Overview of Autobiographical Memories

Scholars analyzed AMs on a wide range of cognitive (e.g., Conway, 2005), clinical (e.g., Berntsen & Rubin, 2007), developmental (e.g., Kulkofsky, 2011), social (e.g., Beaman, Pushkar, Etezadi, Bye, & Conway, 2007; Goddard, Dritschel, & Burton, 1996), and personality psychological studies (e.g., Habermas, 2007; McLean, 2005). People use their AMs as motivations for thinking and talking about their past and to develop a coherent sense of their selves, emotions, future plans, and social relationships with other people (Bluck & Alea, 2008; Conway, 2005; Conway & Pleydell-Pearce, 2000; Habermas & de Silveira, 2008; McLean & Pasupathi, 2006; Olivares, 2010; Pasupathi, 2006). AMs serves three broad functions: directive, self and social (e.g., Bluck, 2003; Cohen, 1998; Pillemer, 1992). The directive-behavior function (i.e., DBF) refers to the use of AMs in guiding present and future thinking and behavior (e.g., Baddeley, 1988; Pillemer, 2003). The self-continuity function (i.e., SCF) regards the method of AM for providing material for the self-concept, and telling people who they are (e.g., Baddeley, 1988; Conway, 1996; Fivush, 1998; Habermas & Bluck, 2000). The social-bonding function (i.e., SBF) relates to the sharing of one's personal memories with others to facilitate social bonding, to stimulate empathy or intimacy, and to communicate or inform (e.g., Alea & Bluck, 2003, 2007; Kulkofsky et al., 2010; Nelson, 1993; Pillemer, 1992). The three AM functions are associated with both measures of detailed recollections (e.g., Rasmussen & Berntsen, 2009) and with dimensions that address the general usage of AMs (e.g., Bluck & Alea, 2009). Several studies examined individual differences in AM functions, showing that individuals who use memories of their relationships to serve social purposes tend to report closer and more satisfying levels of marital satisfaction (Alea & Bluck, 2007; Alea & Vick, 2010); as well, people use the directive function of AMs for guiding their current behavior (Kuwabara & Pillemer, 2010; Kuwabara, Rouleau, & Pillemer, 2011).

## Autobiographical Memories and Personality

Relating AMs to the concept of self (e.g., Brewer, 1986, 1996; Nelson, 1993; Tulving, 2002), previous work assumed a reciprocal association between autobiographical information and personality (e.g., Conway & Pleydell-Pearce, 2000; Habermas & Bluck, 2000; McAdams, 2001; Rubin & Siegler, 2004). Authors proposed that the life story of a person constitutes the most global and abstract level of AMs, encompassing both past and future event representations from the person's entire life (e.g., Bluck & Habermas, 2000; Conway, 2005). Some studies have applied the five-factor model (FFM) of personality (e.g., John & Srivastava, 1999; McCrae & Costa, 1999) to study the relationships between personality dimensions and directive, self and social functions of AMs. The FFM is a well-known approach describing personality variation along five dimensions, called the Big Five, that are respectively named: Extraversion, Openness, Conscientiousness, Neuroticism, and Agreeableness. Extraversion refers to personality traits such as energy, assertiveness, and sociability. Openness regards the tendency to be informed, creative, insightful, curious and to have a variety of experience. Conscientiousness is related to the tendency to be self-disciplined, act dutifully, and aim for achievement. Neuroticism means a tendency to experience unpleasant emotions easily, such as anger, anxiety, depression, or vulnerability. Agreeableness means the tendency to be compassionate, trusting and cooperative rather than suspicious and antagonistic towards others (McCrae & Costa, 1999). Studying the relationships between AMs and personality, Rubin and Siegler (2004) showed that people who are rated high on Openness subjectively experience their AMs with a stronger sense of sensory reliving, vividness, and emotion. As well, Rasmussen and Berntsen (2009) evidenced that Openness correlated positively with the AMs directive and self-functions, while Neuroticism correlated positively only with the self-function. Neither Extraversion, Agreeableness nor Conscientiousness were associated significantly with any of the three functions.

## Autobiographical Memories and Facebook Mementos

Our memory is in a constant mutual interaction process with technologies and social media (Huyssen, 2003). A wide range of research showed not only that individuals who interact with technologies enhance their cognitive skills as, for instance, working-memory (e.g., Caci, D’Amico, & Chiazzese, 2013), but that they also consider social media as virtual storage of transactive memory for their autobiographical self (Lee et al., 2008; Lenhart & Fox, 2006; Wang et al., 2017). Even more often, users' life events are uploaded, for instance, on Facebook and presented, using a short phrase, a picture, a web link to a song or movies, the last book read, the parties attended, the countries visited. Indeed, people collect and share their thoughts, feelings, and activities with friends on their Facebook status (Ryan & Xenos, 2011). Moreover, people use Facebook albums as a repository for photos, recording moments from their lives, or at least a view of life that they wish to portray (Şeyfi & Soydaş, 2017). More specifically, on Facebook textual (i.e., phrases people post on their status) and visual information (i.e., photos and albums) become digital media by which individuals preserve memories about significant people, places or life events in the past (Back et al., 2010; Kalnikaite & Whittaker, 2011; Marshall, 2008; Nosko et al., 2010; Petrelli, Whittaker, & Brockmeier, 2008). The act of posting as well as of uploading media facilitate people to retain and recall their memories (Stevens, Abowd, Truong, & Vollmer, 2003). However, when people use social media, they do much more. As stated by Konrad, Isaacs, and Whittaker (2016), the digital records of people lives, or the so-called technology-mediated memories, facilitate people also to record and reflect upon their life events. While people write about their own experiences in a public and shared sphere, they often receive subsequent social feedback and reflect on the experiences and their relevance.

Consequently, the process of posting online life events that are reflected upon leads people to integrate them into their autobiographical knowledge base and effectively store them for long-term retention. In line with the autobiographical memory literature that highlights the contribution of rehearsal and social sharing to remembering personal experiences (Conway & Pleydell-Pearce, 2000; Nelson & Fivush, 2004; Pillemer, 1998; Wang, 2013), recent studies support the idea that Facebook not only creates a social memory by gathering friendships together but gives users the possibility to collect, remember and share their AMs (Richardson & Hessey, 2009; Wang et al., 2017). A recent study by Seyfi and Soydaş (2017) has analyzed the relationship between AMs and social media usage. More specifically, authors performed ethnographic research on a sample of people, aged between 18 and 50 years, and administered them a semi-structured interview aimed at determining which of the functions of the AMs is influenced by childhood photographs on social media. Results showed that Facebook supports all AM functions offering people a crucial sharing area for present or past events and future purposes.

## Facebook Mementos and Personality

A considerable amount of research has examined the association between textual or visual information people collect on Facebook and personality traits as classified by the FFM (e.g., Amichai-Hamburger & Vinitzky, 2010; Gosling, Augustine, Vazire, Holtzman, & Gaddis, 2011; Ljepava, Orr, Locke, & Ross, 2013; Moore & McElroy, 2012; Ross et al., 2009; Ryan & Xenos, 2011; Seidman, 2013). For instance, in the study by Lee, Ahn, and Kim (2014) extraverted Facebook users were to actively engage in uploading photos and status or wrote comments more frequently than introverted ones. Differently, those scoring high on neuroticism and conscientiousness use Facebook passively. As well, Marshall, Lefringhausen, and Ferenczi (2015) have evidenced that extraverts more frequently updated their Facebook status sharing their social activities and everyday life; whereas, people high in openness were more likely to upgrade about intellectual topics, and those who were high in conscientiousness were more likely to update information about their children. Recently, Dupuis, Khadeer, and Huang (2017) have shown that individuals scoring high on extraversion, agreeableness, and neuroticism are more likely to post positive status updates on Facebook (e.g., job promotion, feeling happy, the birth of a child). As well, individuals scoring low on conscientiousness are more likely to post negative status updates (e.g., feeling lonely, sad, depressed, stressed or angry; disdain for a specific politician and political party). Similarly, Caci, Cardaci, and Miceli (2019) evidenced that extraversion and openness are positive precursors of the amount of information people disclose on Facebook, whereas conscientiousness is a negative one.

## The Present Paper

Starting from the literature mentioned above (e.g., Dupuis et al., 2017; Rasmussen & Berntsen, 2009; Seyfi & Soydaş, 2017), the present study has a twofold goal. The first goal is to empirically analyze the relationships between AM functions, personality traits, and Facebook mementos. We took into account both the number/topics of Facebook status updating (FSU) and the number/contents of photos uploaded on the Facebook by users (i.e., Photos uploading—PU) as objective measures for rating the ways people use the Facebook for recording their AMs (i.e., Facebook mementos). Even if previous studies have agreed about the role of extraversion and openness in promoting the collection and sharing of textual or visual information on the Facebook, they showed inconsistent results for neuroticism and conscientiousness (Dupuis et al., 2017; Lee et al., 2014). Hence, we would verify if personality traits are associated with Facebook mementos (*Hypothesis 1*). More specifically, we tested the predictive role of each personality factors, as defined by the FFM (McCrae & Costa, 1999), both on FSU and PU. Even if the association between personality traits and directive, self and social functions of AMs is not clear (e.g., Rubin & Siegler, 2004; Rasmussen & Berntsen, 2009), we expect to corroborate previous results about the relationships between each of personality traits and the directive, self and social functions of AMs (*Hypothesis 2*). Since people use Facebook for collecting personal reminiscences (e.g., Huyssen, 2003; Kalnikaite & Whittaker, 2011; Petrelli et al., 2008; Seyfi & Soydaş, 2017; Wang et al., 2017), a significant association between measures of all the directive, self and social function of AMs and the number and content of information about life events posted by participants on their Facebook profile was finally expected (*Hypothesis 3*).

The second goal of the present study is to test a model in which personality factors are related to Facebook mementos both directly and indirectly, through the mediation of the directive, self and social functions of AMs. The introduction of mediator variables (i.e., DBF, SCF, and SBF) in the model allows us to deeply understand the relationships between personality traits and textual or visual information people collect on their Facebook profiles (i.e., FSU and PU). Direct paths between personality traits and FSU or PU show whether and how personality dimensions are related to behavioral actions people make during the process of collecting their AMs on Facebook. As well, indirect paths allow us to study the modulation of the relationship between personality and Facebook mementos via the mediation of the functional use of AMs for directive-behavior, social-bonding or self-continuity purposes. Considering that Facebook users collect personal mementos about their lives and share their self for present and future purposes (Seyfi & Soydaş, 2017), the self-continuity function of AMs was expected to mediate this relation more than the directive or social ones (*Hypothesis 4*).

## Method

### Participants

Participants were 193 Italian Facebook users (148 female; 45 male) that voluntarily took part in the study after recruitment via the Facebook page of the researchers' local University. They were mostly undergraduates (82%) aged between 18 and 55 years old (*M*_age_ = 22.8, *SD* = 6.8). All of them were Facebook users, respecting our inclusion criterion for the sample composition. In prevalence, participants were active users of Facebook from more than 5 years (75%); they accessed daily to this social application from three to six times per day (56%) and spent less than 1 hour actively using it (69%).

### Procedure

Due to the goals of the research and considering that we did not gather information related to health or medical issue, data collection procedures did not need to be approved by the Human Research Ethics Committee of the research institution. However, the study followed the international ethical guidelines of the Declaration of Helsinki. Thus, no personal information was collected, and all data were handled according to the Italian law on privacy and the “Ethical Principles of Psychologists and Code of Conduct”. All participants received no monetary compensation or other kinds of reward for taking part in the study. Potential participants had access to a flyer with a brief explanation of the study and a URL link. The flyer was made available on the researchers' University's Facebook page to recruit participants. The link allowed access to the participant information sheet and a confidential online survey via the University's website. All participants provide online consent forms before taking part in the study. Then, they click a “proceed” button, thus starting the online survey, consisting of demographic questions, three questions about their Facebook usage (i.e., *How many years ago did you create your Facebook profile? How many times do you check your Facebook profile per day? How much time do you spend on your Facebook profile per day?*), and the following measures.

### Measures

#### Thinking About Life Experiences Scale-Revised

This 15-item questionnaire by Bluck and Alea (2011) assesses the directive (Directive-Behavior), self (Self-Continuity), and social (Social-Bonding) functions of AMs as individuals' motivations for thinking and talking about the past. It comprises three sub-scales, each consisting of five items related to one of the three functions. Participants rate items on a 5-point scales (1 = rarely, five = very frequently). To ensure fidelity with the original English version of the instrument, we followed the standard guidelines for the process of cross-cultural adaption of self-report measures (Beaton, Bombardier, Guillemin, & Ferraz, 2000). Specifically, we developed an Italian version by process of independent back-translation and discussion with Italian-English bilinguals. For each sub-scale, the total score was computed by averaging the scores obtained by the participants for each of the items of the scale. Cronbach's alpha values in the present study were: 0.74 for Directive-Behavior Function scale (DBF); 0.76 for Self-Continuity Function scale (SCF), and 0.82 for Social-Bonding Function (SBF) scale, and they are quite similar to those reported by Bluck and Alea (2011), in their validation study.

#### Personality Inventory

This 20-item scale questionnaire by Caci, Tabacchi, Scrima, and Cardaci (2014) measures personality in five broad dimensions under the FFM (e.g., Costa & McCrae, 1992). It has five subscales, each consisting of four items related to one of the personality factors: extraversion defined as energy, sociability, and talkativeness; Conscientiousness considered as efficiency and diligence at work; Neuroticism related to emotional instability; Openness concerned with openness to culture and experience, and Agreeableness referred to cooperation with others and trusting. Participants rate items on a 5-point scale (1 = strongly disagree; 5 = strongly agree). For each scale, the total score was computed by averaging the scores obtained by the participant for each of the items of the scale. Cronbach's alpha values of PI in the present study were: .72 for Extraversion; .70 for Conscientiousness; .73 for Neuroticism; .75 for Agreeableness; and, .70 for Openness respectively, in line with those reported by Caci et al. (2014).

#### Facebook Mementos Scale

To have an objective measure of the Facebook mementos, we asked participants to fill the following subscales.

##### Facebook Status Updating

We requested participants to rate both how frequently they update their Facebook status on average per day (1 = Never; 10 = 10 times) and each of the following 18-items related to positive or negative topics of Facebook status update, we derived by Dupuis et al. (2017), using a 5-point Likert scale, ranging from 1 (Never) to 5 (Very often).

Participants answered the question: "*Think back to your status updates on Facebook in the last year. What and whom did it refer, in prevalence?*" To answer, participants referred only to their memory.

Nine items were related to positive topics for status updating: – for example, job promotion or a new job; support and advocacy for a specific politician and/or political party; feeling happy; acceptance into a college/university; a major accomplishment in an academic, sport, or work setting; vacation. The other nine items were associated with negative topics for Facebook status updating – for example, asking for support and/or prayers; feeling lonely, sad, and/or depressed; feeling angry, upset, and/or mad; feeling stressed and/or dealing with a stressful situation; disdain for a specific politician and/or political party; major news events that have an adverse outcome.

##### Photos Uploading

Participants first rated how frequently they upload their photos on the Facebook in a week (1 = Never, 10 = 10 times) and then answered the question: "*Think back to the photo(s) you uploaded to Facebook in the last year. What and/or whom did it depict?*" rating on a 5-point Likert scale, ranging from 1 (Never) to 5 (Very often) each of the following seven topics derived by Houghton, Joinson, Caldwell, and Marder (2013): myself, friend, life events, family, scene, objects, and animals. Also, in this case, participants referred only to their memory to answer.

### Statistical Analysis

**Figure 1:**
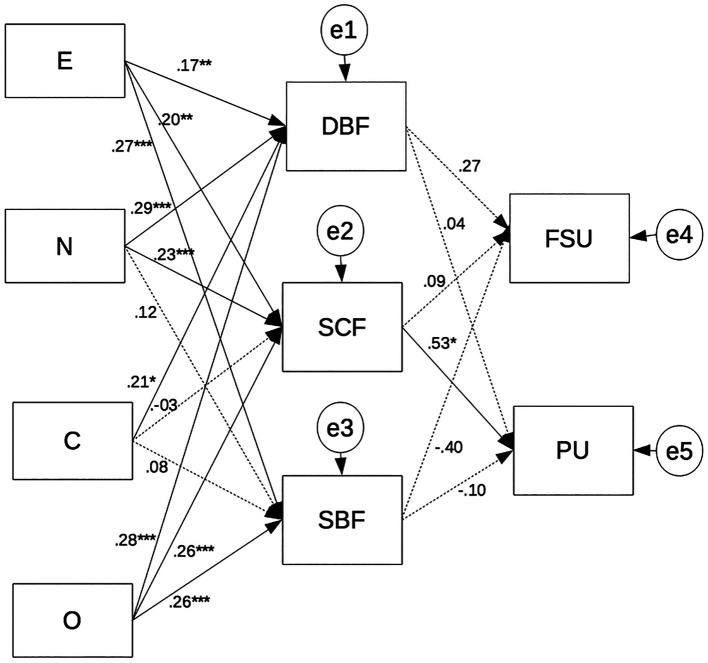
The mediating effect of directive-behavior (DBF), self-continuity (SCF) and social-bonding (SBF) functions of autobiographical memories in associations between personality traits (i.e., E = Extraversion; N = Neuroticism; C = Conscientiousness; O = Openness) and Facebook mementos (i.e., FSU = Facebook Status Updating; PU = Photos uploaded). Direct paths between personality traits and Facebook mementos were removed to improve clarity. Standardized coefficients were presented. Solid lines indicate significant paths at *p < .05; **p < .01; ***p < .01; dotted lines indicate not significant paths at *p* >.05.

To reach our goals, we first calculated descriptive statistics and Pearson's linear correlations using IBM SPSS 20.0 software package (IBM Corp. Released 2011. IBM SPSS Statistics for Macintosh, Version 20.0. Armonk, NY: IBM Corp). Then, to examine whether personality traits were associated with Facebook mementos via the AM directive, self-continuity, and social-bonding functions, we performed a path analysis, using Analysis of Moment Structures (AMOS) software package (Arbuckle, 2014). In line with results of previous studies (e.g., Rasmussen & Berntsen, 2009; Dupuis et al., 2017), we tested a path model consisting of five predictors: Extraversion (E), Neuroticism (N), Conscientiousness (C), Agreeableness (A) and Openness (O); two outcomes: Facebook status updating (FSU) and Photos uploaded on Facebook (PU), and three mediators: Directive-Behavior Function (DBF); Self-Continuity Function (SCF); Social-Bonding Function (SBF). We introduced AMs functions as mediators in the path model to deeply understand the interrelations between personality factors and textual or visual information people store on their Facebook profiles. Direct paths will evidence only the association between personality traits (i.e., the predictors) and Facebook mementos (i.e., the outcomes). *Vice versa*, the introduction of mediators in the model path will allow us to evaluate the specific role of the three different functions of AMs (i.e., DBF, SBF e SCF) in modulating the direct paths (see Figure 1).

## Results

### Descriptive Statistics and Relationships Among AM Functions, Personality Traits, and Facebook Mementos

Table 1 reports the means and standard deviations for the variables of the study. Participants stated they use their AMs more for directive or social-bonding rather than for self-continuity. They describe themselves as extraverts, conscientious, agreeable, and open-minded on average, but with low levels of neuroticism. They update their Facebook status 3.37 times a day, posting mostly FSU related to their feeling of happiness or life events such as acceptance into college/university, a major accomplishment, and vacations. Descriptive data show that participants upload on average 4.58 photos a week, regarding mostly themselves, their friends or their life events.

**Table 1:** Means and Standard Deviations of the Variables of the Study (N = 193)

Variables	*M*	*SD*	Min	Max
AM Directive-Behavior Function^a^	3.40	0.71	1	5
AM Self-Continuity Function^a^	2.63	0.78	1	5
AM Social-Bonding Function^a^	3.33	0.80	1	5
Extraversion^b^	3.27	0.72	1	5
Neuroticism^b^	2.69	0.77	1	5
Conscientiousness^b^	3.48	0.69	1	5
Agreeableness^b^	3.07	0.65	1	5
Openness^b^	3.29	0.62	1	5
Frequency of FSU in a day	3.37	2.28	1	10
Positive FSU Vacations^c^	2.10	1.22	1	5
Positive FSU Job promotion^c^	1.45	0.94	1	5
Positive FSU Weight loss and/or fitness goals^c^	1.22	0.62	1	5
Positive FSU Major Accomplishment^c^	2.15	1.26	1	5
Positive FSU Acceptance into college/university^c^	2.26	1.31	1	5
Positive FSU Feeling happy^c^	2.27	1.26	1	5
Positive FSU Getting engaged^c^	1.43	0.85	1	5
Positive FSU Birth of a child^c^	1.17	0.52	1	5
Positive FSU Support for a politician^c^	1.44	0.85	1	5
Negative FSU Asking for supports (i.e., medical issue)^c^	1.13	0.41	1	5
Negative FSU Feeling lonely/sad/depressed^c^	1.43	0.81	1	5
Negative FSU Feeling stressed^c^	1.53	0.78	1	5
Negative FSU Feeling angry^c^	1.44	0.71	1	5
Negative FSU Mad/angry/upset at a significant other^c^	1.14	0.47	1	5
Negative FSU Having difficulties^c^	1.30	0.59	1	5
Negative FSU News events that have a negative outcome^c^	1.46	0.82	1	5
Negative FSU Mad/angry or upset at a friend^c^	1.21	0.52	1	5
Negative FSU Disdain for a politician^c^	1.42	0.90	1	5
Average number of PU in a week	4.58	2.57	1	10
PU Myself^c^	2.95	1.24	1	5
PU Friends^c^	2.94	1.24	1	5
PU Life events^c^	2.60	1.28	1	5
PU Family^c^	2.18	1.14	1	5
PU Scenes^c^	2.35	1.23	1	5
PU Objects^c^	1.41	0.72	1	5
PU Animals^c^	1.83	1.17	1	5

*Note.* FSU = Facebook Status Updating; PU = Photo Uploading.

^a^AM = Autobiographical Memories. ^b^Personality. ^c^Facebook Mementos.

Table 2 shows Pearson’s zero order’s correlations between Personality traits, Directive-Behavior, Self-Continuity, and Social-Bonding Functions of Autobiographical Memories. We found that DBF and SCF are both positively associated with Neuroticism, *r* = .24, *p* < .01, 95% CI [.10, .36], and Openness, *r* = .31, *p* < .01, 95% CI [.17, .43], whereas, SBF positively correlated with Extraversion, *r* = .27, *p* < .01, 95% CI [.13, .39], and Openness, *r* = .38, *p* < .01, 95% CI [.25, .49].

**Table 2:** Pearson’s Zero-Order Correlations Between Directive-Behavior, Self-Continuity, Social-Bonding Functions of Autobiographical Memories, and Personality (N = 193)

Variables	1	2	3	4	5	6	7	8
1. AM Directive-Behavior Function								
*r*	–							
95% CI	–							
2. AM Self-Continuity Function								
*r*	.48*	–						
95% CI	[.36, .58]	–						
3. AM Social-Bonding Function								
*r*	.48*	.47*	–					
95% CI	[.36, .58]	[.35, .57]	–					
4. Extraversion								
*r*	.18	.18	.27*	–				
95% CI	[.03, .31]	[.03, .31]	[.13, .39]	–				
5. Neuroticism								
*r*	.24*	.22*	.07	-.09	–			
95% CI	[.10, .36]	[.08, .35]	[-.07, .20]	[-.22, .05]	–			
6. Consciensiousness								
*r*	.18	-.03	.13	.06	.27*	–		
95% CI	[.03, .31]	[-.17, .11]	[.01, .26]	[-.08, .19]	[.13, .39]	–		
7. Agreeableness								
*r*	.12	.07	.06	–.05	.07	.08	–	
95% CI	[-.02, .25]	[-.07, .20]	[-.08, .19]	[-.18, .09]	[-.07, .20]	[-.06, .21]	–	
8. Openness								
*r*	.31*	.22*	.38*	.10	.00	.22*	.09	–
95% CI	[.17, .43]	[.08, .35]	[.25, .49]	[-.04, .23]	[-.14, .14]	[.08, .35]	[-.22, .05]	–

*Note.* As suggested by Bujang and Baharum (2016), considering the sample size (*N* = 193), we accepted as significant correlations *r* values above .20, fixing the power to 80% and alpha to .05.

*p < .01.

There were no significant correlations between DBF scores and frequency of FSU textual information posted online both with positive and negative contents. Whereas, significant positive correlations have emerged between DBF scores and frequency of visual media uploaded by participants (i.e., PU), and having contents related respectively to friends, *r* = .23, *p* < .05, 95% CI [.09, .35]; life events, *r* = .30, *p* < .05, 95% CI [.16, .42]; scenes, *r* = .24, *p* < .05, 95% CI [.10, .36], and animals, *r* = .23, *p* < .05, 95% CI [.09, .35]. Similarly, SCF scores not significantly related with the frequency of positive or negative textual information posted on the Facebook status, but significant positive correlations between SCF scores and visual media related to life events, *r* = .31, *p* < .05, 95% CI [.17, .43], and friends, *r* = .23, *p* < .05, 95% CI [.09, .35], have emerged. Positive significant correlations between SBF scores with textual information related to vacations, *r* = .24, *p* < .05, 95% CI [.10, .36], and with visual media related to friends, *r* = .29, *p* < .05, 95% CI [.15, .41], and life events, *r* = .24, *p* < .05, CI [.10, .36], have emerged too. Furthermore, results have shown that people scoring high on extraversion and neuroticism uploaded more frequently photos related to their friends, E: *r* = .24, *p* < .05, 95% CI [.10, .36]; N: *r* = .21, *p* < .05, 95% CI [.10, .34]. People with a high level of conscientiousness tend to post more frequently on their Facebook status textual information regarding their major accomplishment, *r* = .28, *p* < .05, CI [.14, .40], or acceptance into college/university, *r* = .20, *p* < .05, 95% CI [.06, .33]. Participants with high scores on agreeableness uploaded less information related to fitness, *r* = -.20, *p* < .05, CI [-.33, -.06]; whereas those with a high level of openness upload their Facebook status with textual information related both to support for politicians, *r* = .20, *p* < .05, CI [.06, .33], and to news events that have a negative outcome, *r* = .25, *p* < .05, CI [.11, .37]. A complete table with Pearson’s zero-order correlations among Directive-Behavior, Self-Continuity, Social-Bonding Functions of Autobiographical Memories, Personality, and Facebook Mementos variables is provided as supplementary material (Table S1).

### Path Analysis

The indices of fit and path coefficients were examined using maximum likelihood estimates. Path analysis initially performed in a model (Model 1) with all personality factors as predictors (i.e., E, N, C, A, O) two outcomes (i.e., FSU and PU) and three mediators (i.e., DBF, SBF, SCF) revealed bad model fit according to all indicators: GFI = 0.90, AGFI = 0.78, CFI = 0.68, RMSEA = 0.142, 95% CI [0.116, 0.169], χ2 = 112.19, *df* = 23, *n* = 193, *p* = 0.000, χ2/df = 4.87 > 2 (Schreiber, Nora, Stage, Barlow, & King, 2006). Then, considering that no direct associations have emerged between agreeableness and dependent measures, we refined the model and excluded Agreeableness by predictors (Model 2). Path analysis performed on Model 2 with four predictors (i.e., E, N, C, O) two outcomes (i.e., FSU and PU) and three mediators (i.e., DBF, SBF, SCF), revealed a good model fit according to all indicators: GFI = 0.99, AGFI = 0.96, CFI = 0.99, RMSEA = 0.004, 95% CI [0.000, 0.109], χ2 = 4.013, *df* = 4, *n* = 193, *p* = n.s., χ2/*df* = 1.0 < 2 (Schreiber et al., 2006).

Figure 1 shows the standardized path coefficients for Model 2. For the overall sample, results at path analysis showed positive direct paths between Conscientiousness and PU, β = .72, *p* < .05, as well as between Openness and FSU, β = .63, *p* < .05, partially verifying H1. Our data corroborated H2 evidencing significant direct paths between personality factors and mediators (i.e., directive, self and social functions of AMs). Specifically, Extraversion and Openness were positively associated with DBF, E: β = .17, *p* < .01, O: β = .28, *p* < .001; SCF, E: β = .20, *p* < .01, O: β = .26, *p* < .001, and SBF, E: β = .27, *p* < .001, O: β = .26, *p* < .001; Neuroticism was positively associated with DBF, β = .29, *p* < .001, and SCF, β = .23, *p* < .001, and Conscientiousness was positively related only with DBF, β = .21, *p* < .05. The present findings partially verified H3 showing that SCF was directly and positively associated only with PU, β = .53, *p* < .05, but not with FSU. As well, no significant direct effects between DBF or SBF and FSU or PU has emerged.

Finally, we found significant indirect positive paths between personality traits and PU via the mediation of SCF, so corroborating H4. Specifically, high levels of extraversion, neuroticism and openness increase levels of posting photos on Facebook via the mediation of SCF. However, SCF does not mediate the relationship between personality traits and FSU; as well as DBF and SBF do not mediate the relationship between personality traits and FSU or PU. Finally, we found significant indirect positive paths between personality traits and PU via the mediation of SCF, so corroborating H4. Specifically, high levels of extraversion, neuroticism and openness increase levels of posting photos on Facebook via the mediation of SCF. However, SCF does not mediate the relationship between personality traits and FSU; as well as DBF and SBF do not mediate the relationship between personality traits and FSU or PU. We tested the significance of indirect effects using bootstrapping procedures. Standardized indirect effects were computed for each of 1,000 bootstrapped samples, and the 95% confidence intervals were calculated by determining the indirect effects at the 2.5th and 97.5th percentiles. Table 3 reports standardized coefficients and 95% confidence intervals for the structural paths.

**Table 3:** Indirect Effect of Personality Traits on the Number of Photos Uploaded on the Facebook (PU) Mediated by Self-Continuity Function (SCF) of Autobiographical Memories With Associated Bootstrap Standard Errors and Bias-Corrected Confidence Intervals

Personality traits	Effect	SE	95% CI	
			LL	UL
Extraversion	.26*	.006	.003	.26
Neuroticism	.26*	.006	.002	.31
Conscientiousness	.27	.006	-.140	.07
Openness	.30*	.007	.004	.36

*Note*. *SE* = Standard Error; CI = Confidence Interval; LL = Lower Limit; UL = Upper Limit; SEs and CIs based on 1,000 bootstrap samples.

*p < .05.

## Discussion

In the present study, we examined first the relationships between AM functions, personality traits, and Facebook mementos. We evaluated the Facebook mementos rating both the FSU and the PU. Secondly, we tested a path model to evaluate the predictive role of personality traits on Facebook mementos both directly and indirectly, through the mediation of the directive, self and social functions of AMs.

### Personality Traits and Facebook Mementos

Results at Pearson's zero-order correlations in the present study showed that personality traits are related to the ways people use Facebook for recording textual and visual information about their AMs. We found that participants scoring high on extraversion collect and post pictures about their friends on their Facebook profiles as mementos for their autobiographical events. This result is in line with Wang and Stefanone (2013), who reported that extravert Facebook users, who actively engage in Facebook self-presentation, update their status or upload photos more frequently than introverts. Moreover, our data showed that people with high levels of conscientiousness, who usually tend to be devoted to work and family (Wolfradt & Doll, 2001), and are also careful or vigilant on Facebook self-disclosure (Caci et al., 2019; Ozer & Benet-Martínez, 2006), collect positive mementos related both to their familiar or working life events (i.e., vacations, university acceptance or job promotion). Surprisingly, we found that people high on neuroticism also tend to collect photos regarding their friends and upload them to their Facebook profiles. This result is in contrast with literature outcomes regarding the tendency to neurotic people to post fewer photos on Facebook (Amichai-Hamburger & Vinitzky, 2010; Ross et al., 2009; Seidman, 2013). However, young women are a significant proportion of participants in the present study; thus, this outcome might be an effect of gender differences, as evidenced in the literature depicting young women who use Facebook, as more neurotic than man (Amichai-Hamburger, 2013; Correa, Hinsley, & De Zuniga, 2010). It has to be noted that high neurotic young woman also tend to share on Facebook photographs that display essential moments in their lives, or portraying relationships with significant others, such as friends and family (Haferkamp, Eimler, Papadakis, & Kruck, 2012; Huang & Park, 2012; Livingstone, 2008; McLaughlin & Vitak, 2012; Siibak, 2009; Strano, 2008). In the present study, people scoring high on agreeableness collect few mementos on Facebook because of their tendency to be cooperative and optimistic toward the others (Amichai-Hamburger & Vinitzky, 2010; Cervone & Pervin, 2013).

On the contrary, people rating high on openness are more engaged in using Facebook for recording their life events. They update their status more frequently, choose contents more related to ask for support, or advocacy for a specific politician, or to significant news events that have an adverse outcome. Such results are consistent with the literature that describe high openness people as curious individuals, having multiple interests and friends both in their real life and on the Facebook, so tending to disclose and share more information about them, their opinions or their life experiences (Amichai-Hamburger & Vinitzky, 2010; Caci et al., 2019; John & Srivastava, 1999; Seidman, 2013). In sum, results of the present study confirm the role of extraversion, openness, neuroticism and conscientiousness as personality traits that are strongly related with FSU and PU (e.g., Dupuis et al., 2017; Lee et al., 2014; Marshall et al., 2015), showing also specific associations among personality traits and positive or negative topics of FSU and PU.

### Personality Traits and Directive, Self and Social Functions of Autobiographical Memories

The path model tested in the present study evidenced that extraversion and openness are positive precursors of all the three function of AMs (i.e., DBF, SCF, and SBF), whereas, neuroticism positively predict DBF and SCF, and conscientiousness DBF. Such results are partially consistent with previous findings by literature on personality traits and AMs (e.g., Rasmussen & Berntsen, 2009). It is not surprising that people scoring high on extraversion, who generally tend to be sociable, assertive, active, and energetic (McCrae & Costa, 1999), use their AMs to develop, maintain, and enhance their social bonds also on Facebook (Alea & Bluck, 2003; Neisser, 1988; Nelson, 1993; Pillemer, 1998). As well, is entirely comprehensible that people with high levels of openness, who are characterized by intellectual curiosity, creativity and a preference for novelty and variety (John & Srivastava, 1999), might benefit from the DBF of AMs (e.g., King, McKee Walker, & Broyles, 1996; McCrae, 1987; Silvia, 2007, 2008), so collecting on their Facebook profiles many mementos related to their interests. Further association between openness and SCF or SBF we found are also consistent with previous outcomes reported by the literature (Bluck & Alea, 2009; Cappeliez & O'Rourke, 2002; Rasmussen & Bertnsen, 2010). However, the main novelty of the present study regards the predictive role of extraversion both on DBF and SCF of AMs, as well as that of neuroticism and conscientiousness. Starting from the consideration that maintaining existing relationships or creating new friends are two core motives that lead people to develop and use the Facebook (Dunbar, 2016; Gosling et al., 2011; Wang & Wellmann, 2010), we can argue that extrovert people collect their Facebook mementos both for serving directive and self-functions of AMs. Extraverts Facebook users applying the DBF of their AMs use their own experience to construct models that allow them to understand the inner world of the others (i.e., the Friends) and thereby to predict their future behavior (Robinson & Swanson, 1990). Moreover, by applying the SCF function of their AMs, they develop a sense of self-continuity, which provides them with knowledge of their extended self (Neisser, 1988) – that is, a self in the past that can be related to the present self and the projected future self so as to locate themselves across time (Conway, 2005; Conway, Singer, & Tagini, 2004). As well, the “surveillance function” that characterizes Facebook (Joinson, 2008) becomes an enhancing stimulus for nervous and emotionally unstable individuals that, trying to control what is going on online as often as they can, also serve the directive function of AMs. Indeed, they retrieve their past experiences to directing and orienting their future thoughts and behaviors (Baddeley, 1988; Bluck, Dirk, Mackay, & Hux, 2005; Pillemer, 1998), also promoting continuity and development of their identity (e.g., Conway, 1996; Fivush, 1998). Similarly, results from high consciousness individuals, who generally use Facebook purposefully to attain their personal goals (Bachrach, Kosinski, Graepel, Kohli, & Stillwell 2012), support the idea that on Facebook they report past events and the lessons they learned from them as useful in guiding present or future behaviors (e.g., Bluck & Glück, 2004).

### The Directive, Self and Social Functions of AMs and Facebook Mementos

Coherently with our expectation and in line with previous studies (e.g., Huyssen, 2003; Kalnikaite & Whittaker, 2011; Petrelli et al., 2008; Seyfi & Soydaş, 2017; Wang et al., 2017), we found higher significant relationships between directive, self and social functions of AMs and PU. Such results highlight that individual differences on functions of AMs are related to the ways people use the Facebook for recording their life events (e.g., Seyfi & Soydaş, 2017; Wang et al., 2017). Moreover, our findings show that to describe their life events today's young people use SNS more for sharing visual material such as photographs than for writing or posting textual elements (Malik, Dhir, & Nieminen, 2015).

### The Relation Between Personality Traits and Facebook Mementos as Mediated by the Directive, Self and Social Functions of AMs

To our knowledge, the current study is the first to provide empirical evidence for the association between personality traits and Facebook mementos via the mediation of the different functions of AMs. Specifically, we found the linkage between personality traits and PU on Facebook for collecting life events depend on extraversion, neuroticism, and openness and the self-continuity function of AMs mediates it. Even if authors well documented the effect of personality traits on PU (e.g., Eftekhar, Fullwood, & Morris, 2014), it can better be understood in the light of the modulation effect by AMs. Indeed, we can argue that individual differences in personality traits lead people to use Facebook as a new technological medium for collecting and sharing their AMs; in turn, individuals having high levels of extraversion, neuroticism, and openness, are mostly driven by the possibility to use their AMs to experiment a sense of continuity for their self (Zhao, Grasmuck, & Martin, 2008). Finally, during the collection and sharing of photos about their life events, people modulate their past to preserve a sense of being a coherent person over time (Bluck et al., 2008; Fivush, 1998).

### Limitations

Although the research has reached its aims, there are some inherent limitations for interpreting results. First, data comes from an Italian sample; therefore, to generalize the results for larger groups, further studies need to include Facebook users from other countries and cultures. Second, questionnaires of personality traits and directive, self and social functions of AMs adopted self-report measures that, as it is well-known in the literature (Corbetta, 2003), are affected by a social desirability bias. Hence, a social desirability scale should be added to a future version of the questionnaires to reduce this limitation. Third, the Facebook mementos scale, we developed and used in the present study, requested participants to recollect information about the frequency and contents of their Facebook status updating, as well as those about their photo uploading only by memory. Future studies might use measures of information posted online by people, requesting them to look back at their Facebook accounts and to record it. However, in this latter case, privacy concerns need to be taken into account by researchers. Another limitation of the present study is that the path model verified the predictive role of personality factors on objective measures of Facebook mementos through the mediation of directive-behavior, social-bonding and self-continuity functions of AMs without considering the effect of positive vs. negative contents both of the Facebook status updating and of the photo uploading. With concerns to the Facebook status updating subscale we used in the present study, the positive ones that are more related to crucial events of individuals’ life, such as, for instance, a new job, the acceptance into college or the birth of a child, differently from the negative topics referred mostly to feelings or mood state about participants. Hence, further studies are needed to deeply investigate if personality factors and AM functions depend upon the positive or negative topics people posted online as well as if emotion regulation plays a fundamental role. Finally, the prevalence of woman in our sample might be responsible for specific outcomes so that it will be of importance performing future studies aimed at deeply analyzing gender and age differences.

### Conclusion

The present study fills the gap in the literature panorama about individual differences related to personality traits defined in the light of FFM (McCrae & Costa, 1999) and the ways people use the Facebook for recording textual or visual information related to their AMs. We found that personality traits are peculiarly associated both to motivations of using autobiographical memory for the directive, self or social purposes and to visual information people uploaded on Facebook. More specifically, we found that personality traits such as extraversion and openness are positive precursors of the directive, self and social functions of AMs; whereas, neuroticism predicts directive and self-functions of AMs and conscientiousness is a precursor of the directive one. However, only the self-continuity function of AMs modulate the reasons that lead extravert, openness and neurotic people to choose peculiar visual personal mementos to upload (i.e., photos). In this sense, we can argue that people made a personal experience of social media like Facebook using it as repository tool for serving the SCF that give them a sense of being the same person over time while maintaining continuity (e.g., Conway, 2005). Implications of our findings refer to a new way of intending autobiographical memories with the pervasive environments of social media. AMs are continuously constructed and reconstructed by individual and serve an accessible and adaptive function. In turn, social media allow people to retain that personal experiences better. Thus, we can argue that individuals might benefit from this interactive process of posting online, also enhancing the long-term retention of their life events.

## Data Availability

For this study, supplementary materials are freely available (see the Supplementary Materials section).

## Notes

The following supplementary materials are available via the PsychArchives repository (Caci, Cardaci, & Miceli, 2019; https://doi.org/10.23668/psycharchives.2598):

- Table S1, which reports the results of Pearson’s Zero-Order Correlations among Directive-Behavior, Self-Continuity, Social-Bonding Functions of Autobiographical Memories, Personality, and Facebook Mementos Variables (*N* = 193)
